# The Effectiveness of Remineralizing Agents on Dentinal Permeability

**DOI:** 10.1155/2018/4072815

**Published:** 2018-09-12

**Authors:** Marwah Berkathullah, Mohideen S. Farook, Okba Mahmoud

**Affiliations:** ^1^Faculty of Dentistry, University of Malaya, Kuala Lumpur, Malaysia; ^2^College of Dentistry, Ajman University, Ajman, UAE

## Abstract

The effectiveness of remineralizing agents in reducing dentine permeability by tubule occlusion using fluid filtration device functioning at 100 cmH_2_O (1.4 psi) pressure and SEM/EDX analysis were evaluated and compared. Seventy (n = 70) dentine discs of 1±0.2 mm width were prepared from sound permanent human molars. Fifty (n = 50) dentine discs were randomly divided into 5 groups (n = 10): Group 1: GC Tooth Mousse Plus (Recaldent GC Corporation Tokyo, Japan), Group 2: Clinpro™ White Varnish (3M ESPE, USA), Group 3: Duraphat® Varnish (Pharbil Waltrop GmbH, Germany), Group 4: Colgate Sensitive Pro-Relief™ dentifrice (Colgate Palmolive, Thailand), and Group 5: Biodentine™ (Septodont/UK). Dentine permeability was measured after treatment application at 10 minutes, artificial saliva immersion at 7 days, and citric acid challenge for 3 minutes. Data were analyzed by two-way repeated measures ANOVA. Dentine specimens (n = 20) were used for SEM/EDX analyses to obtain qualitative results on dentine morphology and surface deposits. Each treatment agent significantly reduced dentine permeability immediately after treatment application and created precipitates on treated dentine surfaces. All agents increased permeability values after 7 days of artificial saliva immersion except Clinpro White Varnish and Biodentine. Clinpro White Varnish exhibited significant resistance to acid challenge compared to others. Colgate Sensitive Pro-Relief dentifrice has a dual mechanism of action in reducing the dentine sensitivity.

## 1. Introduction

The dentine sensitivity (DS) is a common dilemma in adults' population with a prevalence ranging from 8 to 57% [1] and is ascribed in routine dental practice perceived generally from the second to sixth decade of life [2]. It has a multifactorial etiology and is described clinically as an acute pain caused by excitation of A delta nerve fibers connected to exposed dentine and its tubules due to chemical, thermal, osmotic, evaporative, or tactile stimulation [3]. The dentine exposure to the oral environment perhaps occurs corollary to the gingival recession with tooth surface loss and surgical and nonsurgical periodontal therapies [4]. Dentine sensitivity is the term used to depict the pain experienced by the patient in their formerly insensitive dentine. However, the term dentine hypersensitivity (DH) is ought to be used when clinical manifestations of sensitivity exaggerate compared to the previous history of sensitivity [5].

The hydrodynamic theory as proposed by Brannstrom et al. [6] is a universally accepted theory that provides the presumable explanation for the mechanism of DS. Therefore, the current trend of treatment concentrates on two approaches based on the hydrodynamic theory which are occluding the opened dentine tubules and blocking the activity of dentinal neurons [3]. Advanced exposure to the oral medium perhaps results in the dentine tubule occlusion by the smear layer or pellicle which moderates DH. However, once sensitivity has become established, the pulp is prone to become irreversibly sensitive. Recently, it has been proposed that management should be targeted on not just by achieving the tubule impermeability by its occlusion but also by monitoring the neural components within the pulp to stifle the peripheral stimulatory effects [7].

Numerous physical and chemical treatment agents have been employed till date to manage DH, such as cavity varnishes, calcium compounds, oxalates, strontium chloride, fluoride-based interventions, and lasers. None of these agents in spite of being effective in relieving DH proved to be an ideal [8] as obstructed tubules are generally feeble in contesting regular tooth erosion and abrasion [9].

These therapies are aimed for the dentine sensitivity treatment at present, but the primary challenge is to discover a material or any medication which could best eliminate the pain and does not reoccur which is woefully not employed yet. Formation of insoluble mineral precipitates within the diseased dentinal tubules from the mineralizing agents could be a pragmatic approach to manage the DS. Hence, various approaches in the management have been made to achieve dentine remineralization which eventually forms the significant objective of restorative dentistry [10]. However, there is inadequate indication to confirm the relative efficacy of individual professionally employed treatment agents [11].

Casein phosphopeptide-amorphous calcium phosphate (CPP-ACP) which is a calcium and phosphate-based remineralizing agent acts by providing surplus calcium and phosphate ions into the tooth surfaces containing pellicle and plaque and stabilizing the resultant formed mineral precipitate containing fluoroapatite [Ca_10_(PO_4_)F_2_] [12]. Casein phosphopeptide-amorphous calcium phosphate fluoride (CPP-ACPF) has an addition of 900 ppm of fluoride compared to the former to have an enhanced remineralizing activity by forming mineral precipitates of calcium and phosphate ions with fluoride [13].

Clinpro White Varnish is a remineralizing varnish containing sodium fluoride with innovative tricalcium phosphate. The mechanochemical ball milling process was utilized to modify *β*-tricalcium phosphate using derivatives of carboxylic acid (fumaric acid) [14]. The manufacturer of Clinpro White Varnish has claimed that protective layer which is formed during the process will separate functionalized calcium and fluoride. However, when the varnish contacts the tooth surfaces, the rosin in the varnish dissolves and disintegrates the fluoride, calcium, and phosphate minerals into the oral cavity. These minerals react and form calcium fluoride precipitates which helps in remineralization.

Colgate Sensitive Pro-Relief dentifrice which has a new saliva-based compound containing 8% arginine and calcium carbonate was formulated in the paste form after several investigations that had been carried out to simulate the natural dentine desensitisation behaviour of saliva [15]. The mechanism involved is the deposition of mineral precipitates within the dentinal tubules by the attraction of positively charged arginine towards the negatively charged dentine surfaces. Subsequently, the alkaline environment would enhance the formation of calcium, phosphate, arginine, and carbonate complex leading to remineralization of patent tubules [16].

Biodentine is a calcium silicate-based cement consisting of tricalcium silicate, zirconium oxide, and calcium carbonate in a powder form. Setting reaction of the cement takes place when it is mixed with a liquid composed of water, calcium chloride, and a water-based polymer. It has a shorter setting time and used as a dentine replacement material [17]. The previous study had shown that the hydration process of calcium silicate-based cement would result in the release of calcium hydroxide allowing the formation of biological apatite in the presence of phosphate containing fluids [18]. These potential mineral precipitates could block the diseased tubules of the sensitive dentine. This is the first in vitro study to investigate the efficacy of Biodentine in relieving the dentine sensitivity.

Therefore, the objective of our study was to evaluate the in vitro effectiveness of these remineralizing agents under different conditions simulating the oral environment in the reduction of dentinal permeability. It has become necessary to have studies performed on comparative analysis aiming at its ability to physically plug the dentinal tubules efficiently and decreasing its associated hydraulic conductance, respectively.

## 2. Materials and Methods

### 2.1. Dentine Sample Preparation

The dentine discs were prepared from caries-free human molars extracted for surgical reasons obtained from clinics and multihealth centers under a protocol set by the Ethics Committee of the Faculty of Dentistry, University of Malaya (UM), before the commencement of the study. The teeth were washed with deionized water and then stored in 1% T Chloramines' solution for no longer than 3 months following the extraction before use. Dentine discs (n=70) were prepared by sectioning each molar horizontally with a slow speed sawing diamond disc (Metkon Micracut 125, University of Malaya) under constant water cooling. The coronal and radicular portion were removed from each tooth 1 mm below the occlusal pit and 2 mm below the CEJ [19] to obtain a dentine disc of minimal thickness 1±0.2 mm [20, 21]. The pulp tissue was removed with a barbed broach and care was taken not to damage the predentine surface and the inner wall of the pulp chamber. Samples with exposed pulp horn and calcified pulp chambers were excluded from the study.

**Figure 1:**
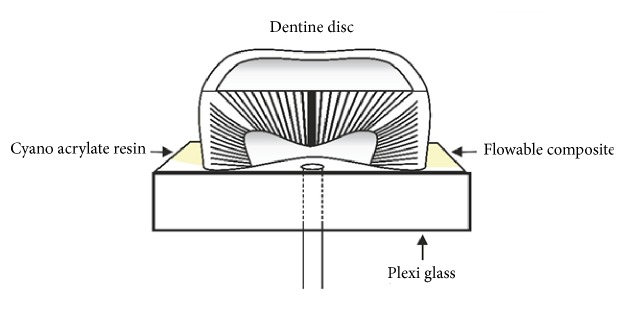
Analogy of a dentine disc attachment to a plexiglass via cyanoacrylate resin and sealing with flowable composite.

The dentine discs were then mounted using cyanoacrylate-based adhesive (Ruichang Dei Adhesive Co., Ltd., China) with the flat radicular side down to an acrylic plexiglass section (YSME Sdn Bhd, Malaysia) which were cut into size 1.5cm x 1.5cm x 0.5cm. The plexiglass was penetrated by a 1.5 cm length of 18-gauge stainless steel tube that permits the pulp chamber to be filled with the fluid. To maintain the tight seal and prevent the leakage, flowable composite (3M ESPE FiltekZ350XT, USA) was applied around the interface between the disc and the adhesive agent as shown in Figure 1.

### 2.2. Fluid Filtration Device

**Figure 2:**
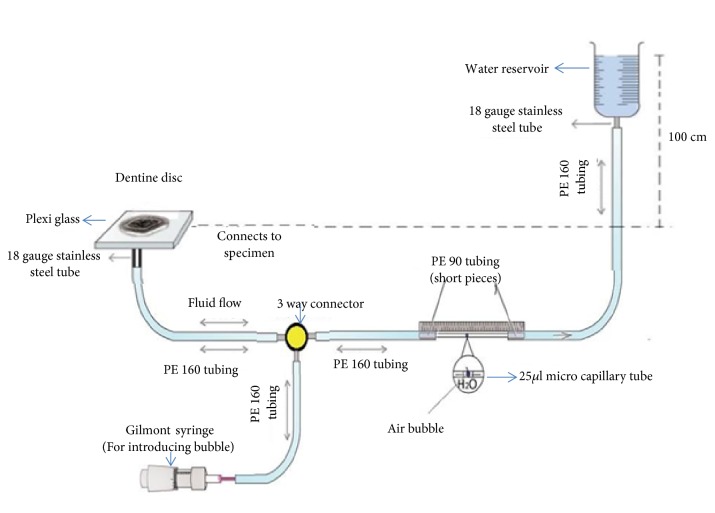
Illustration of an experimental set-up depicting ‘'fluid filtration device”.

The quantitative changes in the dentine permeability (Lp) through the dentine disc specimens induced by the treatments (T) applied were quantified by fluid filtration device (Figure 2) working at 100 cm H_2_O (1.4 psi) pressure [22]. Each dentine discs were connected to a water-filled system using polyethylene (PE) tubes. A 25 *μ*m microcapillary tube (Microcaps, Fisher Scientific, Atlanta, GA, USA) was horizontally positioned between the water reservoir and the dentine disc. A Gilmont syringe (Thermo Scientific, USA) was used to insert and control the position of the air bubble within the device.

## 3. Experimental Design

### 3.1. Permeability Measurements for Treated Dentine

**Figure 3:**
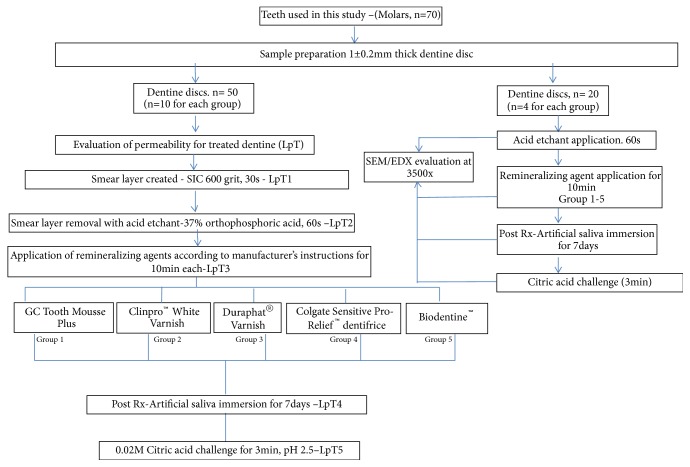
Summary of the experimental design for measuring permeability of dentine treatments (LpT) and SEM/EDX analysis.

Ten samples for each group were allocated randomly (n =50) for groups 1-5. The fluid flow through the dentine disc was measured after each stage of treatment.  Figure 3 shows the summary of the experimental design for dentine permeability measurement of different treatments (LpT).

The upper dentine surface was wet sanded with 600 silicon carbide abrasive grit paper for 30 seconds to create a standard smear layer. This established a minimum permeability value for the first treatment (LpT1). Then, the dentine surfaces of the discs were treated with acid etchant, 37% ortho phosphoric acid (3M7423 Scotchbond etchant), for 60 seconds and washed with distilled water. This established a maximum permeability value for the second treatment (LpT2). Subsequently, dentine discs were subjected to five different remineralizing agents by following the manufacturer's instructions of the materials for 10 minutes each and its values were measured (LpT3). The active ingredients of the materials that were used are summarized in Table 1. Fourth treatment (LpT4) values were obtained by immersing the treated dentine disc in artificial saliva at 37°C (pH7.4) for 7 days. The composition of artificial saliva (Alfatech Sdn Bhd, Malaysia) was CaCl_2_ 1.5mmol/L, KCl 50 mmol/L, KH_2_PO_4_ 0.9 mmol/L, and Tris 20 mmol/L [23]. The final value (LpT5) was recorded by challenging the dentine discs to 0.02 M citric acid solution (pH 2.5) for 3 minutes.

The linear movement of a bubble was observed against a calibrated ruler incremented and measured in mm and converted into volume displacement (hydraulic conductance, Lp) pertaining to dentine permeability. It was measured for 4 minutes with 3 consecutive measurements after the system had been stabilized for a minute. The dentine permeability for treated dentine (LpT) was calculated by dividing the fluid flow (*μ*L) by surface area of dentine exposed (cm^−2^) and hydrostatic pressure cm H_2_O/psi. Each dentine specimen permeability was presented as percentage (LpT%) of the hydraulic conductance across acid etched dentine disc of the same specimen. Therefore, each specimen acted as its own control.

### 3.2. SEM/EDX Analysis

Dentine discs (n=20) were prepared following the specimen preparation method used for dentine permeability measurement. Four dentine discs amongst those were selected randomly, prepared, and processed for the qualitative analysis of each treatment group for 60 seconds of acid etching, application of remineralizing agents for 10 minutes according to manufacturer's instructions, artificial saliva immersion for 7 days, and citric acid challenge for 3 minutes. Each dentine discs were air dried in a sun-dry cabinet at a constant room temperature 37°C and sputter coated with gold in a vacuum evaporator (Polaron Q150RS, Hi-Tech Instruments Sdn Bhd, Malaysia).

The scanning electron microscope (SEM) (Quanta FEG 250, Crest Co., Holland) with a low vacuum operating mode was used to examine the specimens at 10 KV and subjected to morphological analysis. The SEM images of each specimen were captured using the magnification 3500x. This SEM is also equipped with an energy X-ray dispersive spectrometer-EDX (Oxford x-Max using software Inca, Crest Co., Holland) which was used for elemental and chemical analysis of dentine samples. All the samples were analyzed at 20 KV. This was done to see the tubule occlusion, crystal precipitates, and the elements present. The protocol for SEM/EDX analysis is shown in Figure 3.

### 3.3. Statistical Analysis

Statistical analysis was performed using SPSS version 20 for Windows. The permeability data (LpT1, LpT2, LpT3, LpT4, and LpT5) were transformed into percentages of the original LpT2 (maximum permeability) values. Means and standard deviations of LpTs values were calculated for each group. Homogeneity of variance was assessed by using Levene's test (p > 0.05). Two-way repeated measures ANOVA procedure was used to evaluate intertreatment and intratreatment effects. Post hoc multiple comparisons were used to identify significant differences (p < 0.05) between the groups using Bonferroni test. All* p*-values were set at < 0.05.

## 4. Results

### 4.1. Permeability Measurements for Treated Dentine

All treatments showed a decrease in dentine permeability. The changes in the dentine permeability produced by the application of different therapies are presented in Table 2.

Dentine permeability after acid etching treatment (LpT2) increased the permeability to a maximum level equal to 100% (arbitrary value). Each acid etched dentine represented its own control of that specimen [24]. The application of all the remineralizing agents on the acid etched dentine resulted in significant reduction of permeability (p < 0.05) compared to its maximum acid permeability value. However, no statistically significant differences were observed amongst all the treatment groups.

Surprisingly, immersion of treated specimens in artificial saliva for seven days (LpT4) resulted in an increased mean dentine permeability values for all the agents except for Clinpro White Varnish. It showed significant differences in the reduction of dentine permeability (p < 0.05) compared to other remineralizing agents amongst the group excluding Biodentine. The exposure of treated dentine specimens to citric acid led to an increase in dentine permeability values of all treatment groups. However, Clinpro White Varnish showed statistically significant resistance to citric acid challenge compared to other remineralizing agents (p < 0.05).

### 4.2. Dentine Morphological Evaluation by SEM/EDX Analysis

**Figure 4:**
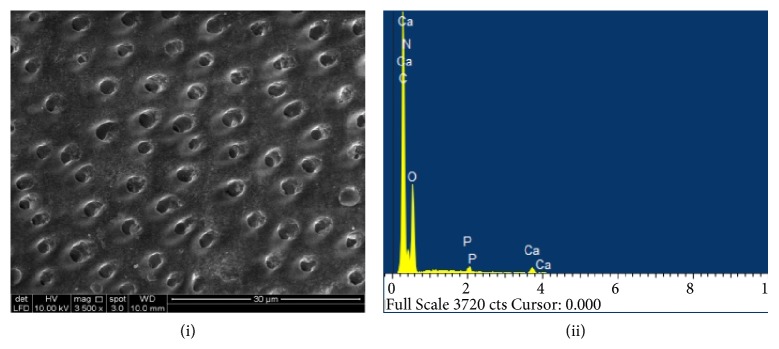
(i) SEM image (3500x) and (ii) EDX spectra of acid etched dentine specimen. Dentine surface exhibits the complete patency of dentine tubules following the acid etchant application. EDX analysis revealed low peaks of Ca and P.

The process of remineralization with occlusion of dentinal tubules was investigated further by using scanning electron microscopy and element sensitive detector to record qualitatively the elemental composition that is Ca, P, and other elements within the treated dentine specimen which is by the experimental protocol [25]. Dentine discs (n=4) were subjected to SEM/EDX analysis immediately after acid etching which led to the complete wear-out of smear layer and exposure of dentine tubules. The EDX spectra demonstrated mineral deficient dentine surface with reduced mineral peaks and an increase in N, O, and C (Figure 4).

**Figure 5:**
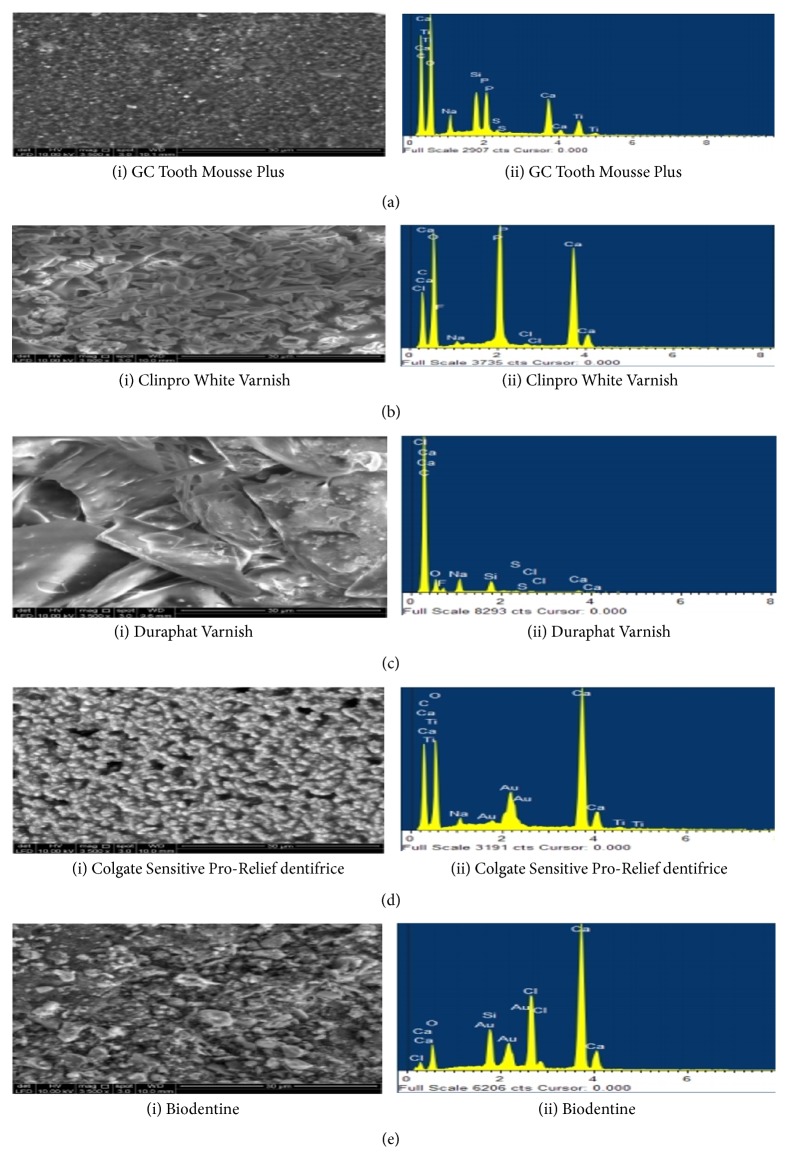
SEM images (3500x) and EDX spectra of dentine surfaces following the application of remineralizing agents for 10 minutes. (a) The surface of acid etched dentine samples treated by GC Tooth Mousse Plus demonstrated layers of granular deposits with a cobble stone appearance occluding dentinal tubular orifice with few vacant tubular orifices visible. EDX spectra revealed the prevalent content of Ca and P. (b) Clinpro White Varnish on dentine surface resulted in dentine tubule occlusion covering the entire dentine surface with a fine hexagonal crystal layer, whereas EDX spectra revealed high content of Ca and P. (c) Duraphat Varnish application created a finely crystalline surface masking all the dentine tubule orifices. No open tubule orifices were observed. Low levels of Ca were observed on EDX analysis. (d) Colgate Sensitive Pro-Relief dentifrice application produced a layer of finely coarse granular precipitates masking the dentine surface but only few open tubules were apparent. EDX analysis revealed high content of Ca. (e) Biodentine demonstrated different size and shape of mineral particles covering the entire surface of dentine leaving few vacant orifices of dentinal tubule. EDX analysis revealed high content of Ca.

**Figure 6:**
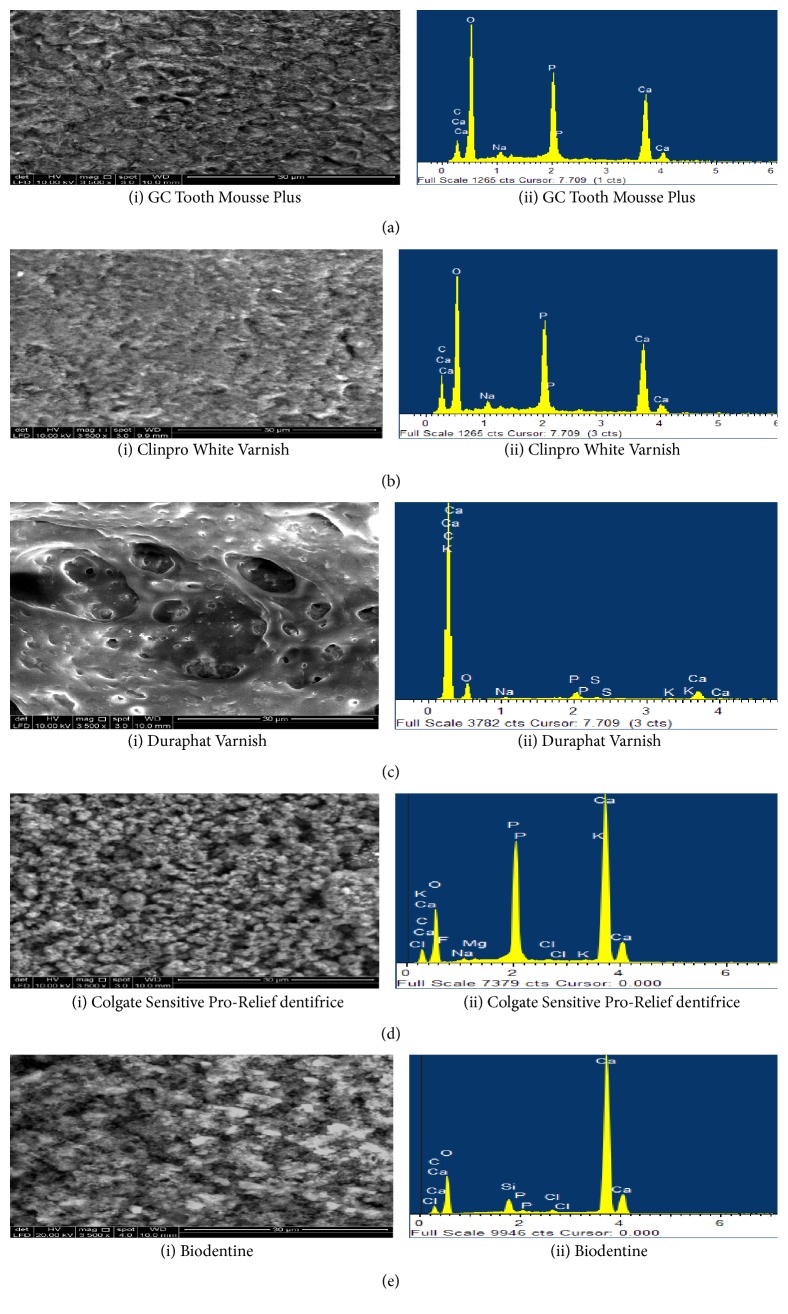
SEM images (3500x) and EDX spectra of treated dentine specimens immersed in artificial saliva for 7 days. (a) GC Tooth Mousse Plus demonstrated modifications in the particles morphology with the deposits being partially removed from the tubules were observed. EDX detected high peaks of Ca and P. (b) Clinpro White Varnish demonstrated a homogenous surface with less partially blocked tubular orifice, whereas EDX revealed high peaks of Ca and P. (c) Duraphat, the surface appeared smooth with only few deposits and nonsignificant closures of tubules were observed, whereas EDX analysis revealed low levels of Ca and P elements. (d) Colgate Sensitive Pro-Relief dentifrice resulted in drastic change of its morphology with much hazy appearance with sparse tubules obliterated by the mineral precipitates. EDX detected high peaks of Ca, P, and K. (e) Biodentine, dentine surface appeared to have a globular morphology with further slight increase in the number of exposed dentinal tubules. An increase in Ca content was observed on EDX analysis.

**Figure 7:**
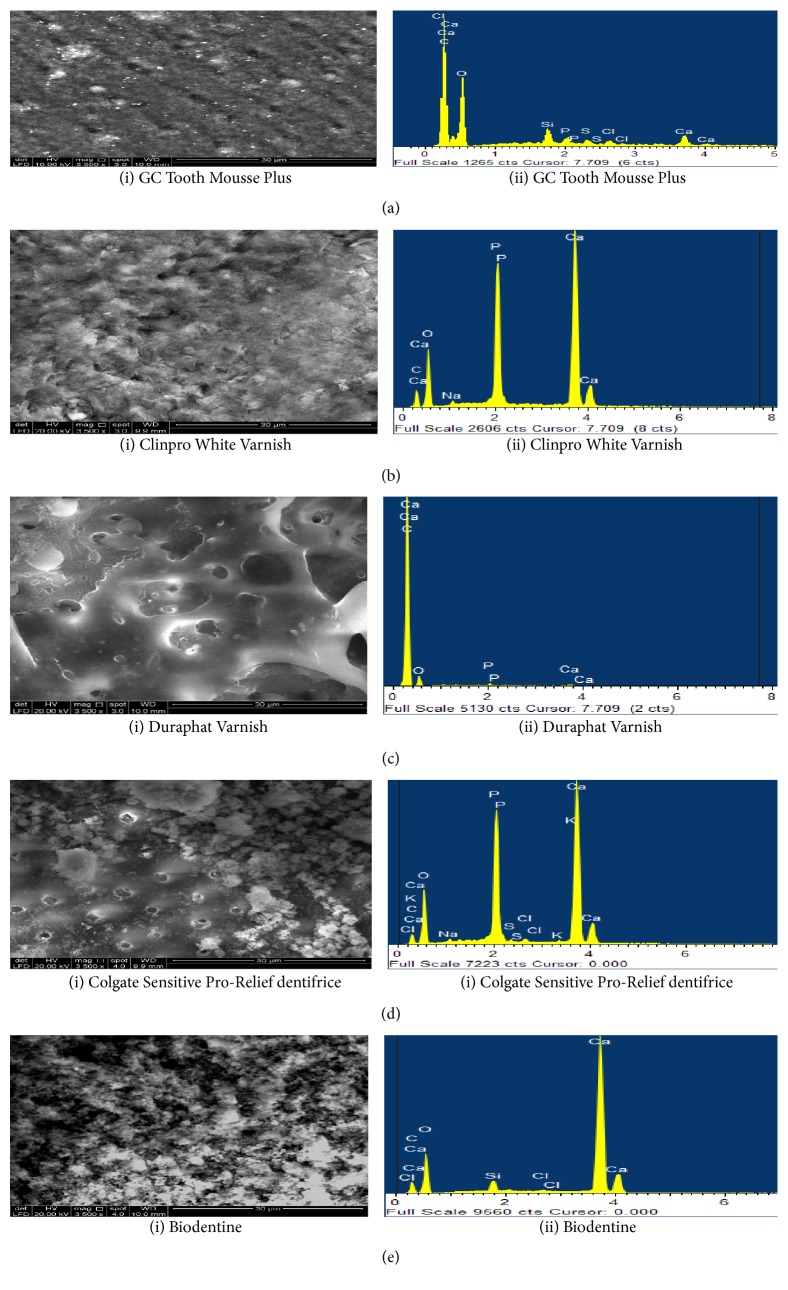
SEM images (3500x) and EDX spectra of treated dentine specimens after 3 minutes of citric acid challenge. (a) Deposits of GC Tooth Mousse Plus were eroded away leading to an increased exposure of dentinal tubules and an abrupt decrease in the content of Ca and P in the EDX analysis (b) Clinpro White Varnish resulted in a much smoother and uniform morphology exhibited with few unoccupied dentinal tubules. The crystal-like structures were rarely evident. EDX analysis revealed the peaks of Ca and P which were still maintained. (c) Duraphat resulted in the removal of tubular plugs and exposure of the orifices. EDX analysis revealed low levels of Ca and P elements. (d) Dentine surface treated with Colgate Sensitive Pro-Relief dentifrice appeared to be washed out and revealed the intertubular dentine with few tubules partially plugged. There were not many changes to the high Ca and P peaks in EDX spectra. The element K was evident during this stage. (e) Biodentine resulted in an increase in the number of vacant dentinal tubules, whereas EDX showed that Ca remains unchanged even after being challenged to citric acid with the presence of Si.

SEM analysis of acid etched dentine samples treated with GC Tooth Mousse Plus demonstrated layers of granular deposits with a cobblestone appearance. The surface appeared to be homogeneously covered occluding the dentinal tubular orifice. Only a few orifices of dentinal tubules were visible. EDX spectra revealed the prevalent content of Ca and P (Figure 5(a) (i) and (ii)). After seven days of immersion in artificial saliva, modifications in the particles morphology with the deposits being partially removed from the tubules were observed. Additionally, EDX detected high peaks of Ca and P (Figure 6(a) (i) and (ii)). The subsequent challenge to citric acid solution further eroded away the deposits leading to increased exposure of dentinal tubules and an abrupt decrease in the content of Ca and P in the EDX analysis (Figure 7(a) (i) and (ii)).

The application of Clinpro White Varnish on dentine surface resulted in dentine tubule occlusion covering the entire dentine surface. A fine hexagonal crystal layer was observed and plugged the dentinal surface at treatment application. EDX analysis had revealed the high content of Ca and P (Figure 5(b) (i) and (ii)). The artificial saliva immersion for seven days had demonstrated a homogenous surface with less partially blocked tubular orifice where high peaks of Ca and P were observed under EDX spectra (Figure 6(b) (i) and (ii)). A much smoother and uniform morphology was exhibited after the citric acid challenge for 3 minutes with few unoccupied dentinal tubules. The crystal-like structures were rarely evident. However, the peaks of Ca and P are still maintained the same level even after the citric acid challenge under EDX analysis (Figure 7(b) (i) and (ii)).

Duraphat Varnish treatment application created a finely crystalline surface masking all the dentine tubule orifices. No open tubule orifices were observed (Figure 5(c) (i) and (ii)). However, after seven days of immersion in artificial saliva, the surface appeared smooth with only a few deposits and nonsignificant closures of tubules were observed (Figure 6(c) (i) and (ii)). Subsequently, the citric acid challenge for 3 minutes further led to the removal of tubular plugs and exposed the orifices (Figure 7(c) (i) and (ii)). Furthermore, it showed low levels of Ca and P elements during all the stages of treatment under EDX analysis (Figures 5(c)(ii), 6(c)(ii), and 7(c)(ii)).

Colgate Sensitive Pro-Relief dentifrice treatment application produced a layer of finely coarse granular precipitates masking the dentine surface but only a few open tubules were apparent. EDX analysis after the treatment applied to the dentine surface revealed the high content of Ca (Figure 5(d) (i) and (ii)). The following immersion in artificial saliva for seven days resulted in a drastic change of its morphology with thick and smooth surfaces being observed. Apparently, sparse tubules were obliterated by the mineral precipitates (Figure 6(d) (i) and (ii)). Further with the citric acid challenge for 3 minutes, the surface appeared to be washed out and revealed the intertubular dentine. Few tubules were partially plugged inevitably (Figure 7(d) (i) and (ii)) and there were not many changes to the high Ca and P peaks for both artificial saliva immersion and citric acid challenge in EDX spectra. However, the element K was also observed to be consistently present in the latter two stages of dentine treatments (Figures 6(d)(ii) and 7(d)(ii)).

Biodentine applied to the dentine surface demonstrated different size and shape of mineral particles covering the entire surface of dentine leaving few vacant orifices of the dentinal tubule. EDX analysis of treatment application demonstrated high content of Ca (Figure 5(e) (i) and (ii)). However, following the artificial saliva immersion for seven days, the dentine surface appeared to have a globular morphology with a further slight increase in the number of the exposed dentinal tubules. Furthermore, there was an increase in Ca content when immersed in artificial saliva for seven days (Figure 6(e) (i) and (ii)). The subsequent citric acid challenge to the dentine specimen increased the number of vacant dentinal tubules. However, EDX had shown that Ca remain unchanged even after being challenged to citric acid with the presence of Si (Figure 7(e) (i) and (ii)).

## 5. Discussion

Dentine hypersensitivity is a persistent and a troublesome clinical malady which at times is misdiagnosed by dental experts who perhaps struggle to resolve the problem to their patients' satisfaction efficaciously [26]. The hydrodynamic theory is a universally accepted theory that best explains the mechanism of dentine hypersensitivity, and hence, any material that results in a decrease of dentine permeability by occluding tubules would eventually be capable of reducing the symptoms of dentine hypersensitivity clinically [27]. The assessment of apatite and crystal mineral precipitate deposition in stages when immersed in virtual body fluids is a generally accepted means to ascertain the bioactivity of a component to remineralization. A key factor essential to decrease hypersensitivity is the hydraulic conductance method which provides the quantitative data illustrating the potential of occluding precipitate to impede the outward fluid flow through dentine tubules. Sealing of the tubules can be obtained by physical occlusion via particles, stimulation of natural mineral, hydroxycarbonate apatite formation above and within the tubules, or a combination of the two [28].

Therefore, the present study was designed to investigate the bioactive characteristics of potential remineralizing agents to decrease dentine permeability over seven days of artificial saliva immersion with following citric acid immersion for 3 minutes through tubule occlusion. This fluid filtration device has been used for the in vitro assessment of remineralizing agents by dentine permeability in various studies which was developed by Pashley and his colleagues [29].

The applied pressure is a variable which is not apparently standardized and has been reported with no justification and comparison to the physiological pulpal pressure [30]. Various pressures such as 20 cm H_2_O, 70 cm H_2_O, and other higher pressures have been used in dentine permeability studies. However, a study had concluded that higher mean permeability value was achieved using 100 cm H_2_O pressure compared to 14 cm H_2_O pressure. Therefore, the author suggested using 100 cm H_2_O pressure for this fluid filtration device measuring the dentine permeability [31].

The study regarding the efficacy of GC Tooth Mousse Plus to decrease dentine permeability is very scarce. The result of the present study had shown that the low concentration of fluoride with casein phosphopeptide-amorphous calcium phosphate has less benefit in reducing dentine permeability compared to high concentration fluoride Duraphat Varnish. Although it had reduced the dentine permeability during initial application, the subsequent dentine treatments showed increase in permeability to a greater extent compared to other remineralizing agents. Increased dentine permeability after immersion in artificial saliva for a week was evident from the SEM analysis showing patent tubules. A high Ca and P peak in the LpT4 stage has indicated the mineralizing potential of the agent. However, an abrupt drop of both the elements after the citric acid challenge has denoted the mineral precipitates which were formed is not strong enough to withstand the acid challenge. Based on the results, it could be assumed that this agent requires several applications to be effective in reducing the sensitivity. It is hard to accept that single application can form a stable mineral precipitate within the dentine tubules.

Clinpro White Varnish is a functionalized tricalcium phosphate technology which is formulated with sodium fluoride (fTCP) and is known to remineralize enamel subsurface lesions. The results show that it is the only agent amongst the group which reduced the dentine permeability after one-week immersion in artificial saliva, while the other treated specimens increased the dentine permeability during the same stage. This would perhaps be due to the mineral precipitates formed within the tubules which were stabilized and are hard to be dislodged out from the tubules by the pressure exerted by the filtration device. Additionally, it showed significant resistance to the citric acid challenge compared to other agents. It has been reported in a study that the higher the negative solubility constant (Ksp), the lower the solubility of ions. The Ksp of tricalcium phosphate at 25°C is reported to be 2.7 × 10^−33^, which could be the reason it was capable of withstanding citric acid challenge compared to other remineralizing agents [32]. This finding is supported by EDX spectra showing stable Ca and P elements for both LpT4 and LpT5 stages. The SEM evaluation demonstrates significantly minimal opening of dentinal tubule orifices during the last three levels of permeability analysis.

Duraphat Varnish has been previously used in some studies and is well known therapeutic agent of dentine hypersensitivity. It is one of the most common desensitisers used for dentine sensitivity and is known to reduce the sensitivity by occlusion of tubules via crystallization and to decrease dentinal fluid flow to the pulp [33]. In this study, the results demonstrated that immediately after treatment application, permeability was reduced significantly, but there was an increase in permeability after artificial saliva immersion for a week which suggests perhaps that the artificial saliva medium was aggressive to dissolve the unstable mineral components and to expose the tubules as evident by SEM analysis. The EDX spectra also indicate that the element F (Fluoride) was hardly detected with little presence of other minerals in that treatment stage. Subsequently, its results failed to show excellent resistance to the citric acid challenge because of increased permeability values. The appearance of patent dentinal tubules in SEM images and reduced mineral contents in EDX analysis concludes the ineffective nature of agent which is susceptible to acid challenge. This could be attributed to the lower negative solubility constant of CaF ions (1.46x10^−10^) where calcium fluoride in the presence of acids forms calcium and fluoroapatite which could be dissolved in acidic pH [32]. A clinical study by Hansen et al. had demonstrated that the application of sodium fluoride varnish had relieved more than fifty percent of teeth with dentine hypersensitivity [34], while other in vitro studies including the current study showed the poor efficacy of fluoride in reducing dentine permeability [35]. Literature shows conflicting reports of fluoride on its actions and effects in dentine sensitivity treatment.

Colgate Sensitive Pro-Relief dentifrice contains arginine and calcium carbonate that functions in conjunction to speed up the natural obliteration mechanism of dentine tubules by forming dentine like minerals containing calcium and phosphate [36]. The treatment application for this agent had shown a dentine permeability reduction of 68% which is similar to the study done by Sauro et al. [37]. High Ca peaks in EDX spectra were observed at treatment application which possibly is due to the formation of calcium-containing precipitates covering almost all the entire dentinal surfaces and occluding dentine tubules in SEM. There was an increase in the dentine permeability to 10% after immersion in artificial saliva for a week with few openings of dentinal tubules in SEM that would suggest that this factor is due to the dislodgement of some weak precipitates from the tubules due to the hydrostatic pressure. However, the emergence of P and K elements in EDX could be due to the interaction of treatment agent with artificial saliva resulting in the remineralization. This is consistent with the ideal mechanism where arginine and calcium carbonate react in the presence of saliva to deposit calcium and phosphate minerals. The dentine permeability had further increased to 17% after citric acid challenge. This result agrees with the SEM showing the particles were washed away from the surface and exposing the tubules. Sauro et al. had reported that 47% of the dentinal tubules had resisted the citric acid challenge [37]. Similarly, this study had shown that the resistance of the tubules to the acid challenge was 42% by this remineralizing agent. Another interesting finding of the study was the presence of K peak in LpT4 and LpT5 stages. This element K can act as nerve stabilizer in patients with dentine sensitivity. It can be concluded that Colgate Sensitive Pro-Relief dentifrice has dual actions in managing the dentine sensitivity occluding the open tubules and inhibiting the excitation of intradental nerves.

Biodentine is calcium-based silicate cement and has been proposed by a study that this cement could occlude dentinal tubules with stable and acid resistant calcium phosphate deposits [38]. Biodentine had behaved similarly as that of another remineralizing agent in reducing the permeability at treatment application stage. Subsequently, it does not show any significant differences in permeability reduction compared with Clinpro White Varnish in the LpT4 stage. Dentine permeability reduction was more than 50% after citric acid challenge. Values of all the permeability stages are consistent with the results of SEM. The number of opened tubules gradually increased from treatment application stage to citric acid challenge. EDX analysis after treatment application, storage in artificial saliva for a week, and eventual treatment with citric acid had demonstrated high Ca peak with Si indicating the presence of key minerals such as calcium and silica involved in the reduction of permeability in all the experimental stages. Additionally, the presence of element P generated during immersion in artificial saliva is from the precipitates of Ca and P minerals. This could be a reason why it was demonstrating significant dentine permeability reduction comparable to Clinpro White Varnish during the particular stage. A study by Gandolfi et al. also showed the similar EDX findings when they used a material derived from calcium silicate-based cement [38].

## 6. Conclusions

Overall, all the remineralizing agents were able to occlude exposed dentine tubules and reduce the dentine permeability in the presence of simulated oral environment. Clinpro White Varnish significantly decreased permeability through tubule occlusion and exhibited excellent remineralizing potential. Additionally, it showed marked resistance to the acid challenge. Colgate Sensitive Pro-Relief dentifrice has a dual mechanism of action in reducing the dentine sensitivity.

## Figures and Tables

**Table 1:** Active ingredients of remineralizing agents applied.

**Remineralizing agents**	**Manufacturer**	**Active Ingredients**
GC Tooth Mousse Plus	Recaldent GC Corporation Tokyo, Japan.	10% CPP-ACP and 0.2% sodium fluoride

Clinpro™ White Varnish	3MESPE, USA	Tri-calcium phosphate, 5% sodium fluoride

Duraphat® Varnish	PharbilWaltrop GmbH.Waltrop, Germany.	5% Sodium fluoride

Colgate Sensitive Pro-Relief™ dentifrice	Colgate –Palmolive, Thailand	8% Arginine and 1.1% sodium monofluorophosphate

Biodentine™	Septodont / UK	Tri-calcium silicate, di-calcium silicate, calcium carbonate and oxide filler

**Table 2:** Permeability (Lp) data after treatments at different Lp stages.

Treatments (Lp%)	GC Tooth Mousse Plus (Group 1)	Clinpro™ White Varnish (Group 2)	Duraphat® Varnish (Group 3)	Colgate Sensitive Pro-Relief™ dentifrice (Group 4)	Biodentine™ (Group 5)
Acid etchant application- LpT2	100±0	100±0	100±0	100±0	100±0
Treatment application- LpT3	32.8±15.8a	28.4±13.4a	30.7±17.9a	31.8±13.1a	30.7±19.7a
Artificial saliva immersion- 7 days- LpT4	44.7±11.3b	20.2±7.6c	44.5±19.6b	41.7±14.8b	34.4±12.1abc
Citric acid challenge- 3minutes LpT5	66.2±7.5d	27.5±11.7a	60.7±14.7d	58.0±16.8d	54.3±15.4d

The values expressed as (%) were reported as means ± standard deviations (n=10) per group identified by different alphabets which are significantly different. All p values are less than 0.05 (p <0.05).

## Data Availability

The data used to support the findings of this study have not been made available because the data is confidential and has to be within the premises of University of Malaya. Therefore, the data would not be available upon request.

## Abbreviations

DS: Dentine sensitivity
DH: Dentine hypersensitivity
SEM: Scanning electron microscopy
EDX: Energy dispersive X-ray
Lp: Permeability.
